# Diet and Cardiovascular Risk in University Marching Band, Dance Team and Cheer Squad Members: a cross-sectional study

**DOI:** 10.1186/1550-2783-5-9

**Published:** 2008-04-18

**Authors:** Shreela V Sharma, Jill A Bush, Andrew J Lorino, Mark Knoblauch, Diana Abuamer, Gabe Blog, Dave Bertman

**Affiliations:** 1 Division of Epidemiology, Michael and Susan Dell Center for Advancement of Healthy Living, The University of Texas Health Science Center at Houston, School of Public Health, Houston, USA; 2 Department of Health and Human Performance, The University of Houston, Houston, USA; 3 Moores School of Music, The University of Houston, Houston, USA

## Abstract

**Background:**

Cardiovascular disease (CVD) is the leading cause of death in the United States. Diets high in fat, especially saturated fat, are often linked to obesity, hypertension and hypercholesterolemia, all risk factors for CVD. The purpose of this study was to determine the association between diet and CVD risk factors in members of a university marching band, dance team and cheer squad.

**Methods:**

In 2004, 232 marching band, dance team and cheer squad members completed a self-administered survey evaluating dietary intake. Body mass index (BMI), waist-to-hip ratio (WHR), blood pressure, fasting serum glucose and cholesterol were measured. Unpaired t-test and Pearson's chi square test were used to determine baseline differences by gender. Multiple linear regression analysis was used to determine the cross-sectional association between dietary intake of various food groups such as grains, meats, fruits & vegetables, dairy, water, alcohol and risk factors for CVD namely BMI, WHR, blood glucose, total cholesterol, and blood pressure (BP).

**Results:**

45% of the participants were overweight; 30% of females and 4.3% of males had WHR ≥ 0.80 and 0.95 respectively. Almost 8% were hyperglycemic, 10% hypercholesterolemic, 15% had high systolic and 9% had high diastolic BP. Less than 50% consumed the recommended servings of grains, fruits and vegetables, dairy and water and 58% consumed alcohol. Higher grains intake was positively associated with higher BMI (Adjusted β = 1.97, p = 0.030, 95% CI: 0.19, 3.74) and; higher alcohol intake was also positively associated with higher BMI (Adjusted β = 0.15, p = 0.002, 95% CI: 0.06, 0.24).

**Conclusion:**

These results warrant the evaluation of existing college-based health programs and development of new interventions to improve dietary habits and promote a healthy lifestyle in these athletes.

## Background

Dietary habits are typically developing in childhood and established by young adulthood [1]. Development of healthy eating habits in young individuals could be vital for prevention of cardiovascular disease (CVD) since CVD is the leading cause of death in the United States [2]. Risk factors for CVD include obesity, hypertension, hypercholesterolemia, insulin resistance, physical inactivity, and smoking [3] and recent data have shown a significant rise in the prevalence of obesity in children, adolescents and adults [4]. Diets high in fat, especially saturated fat, are often linked to obesity, hypertension and hypercholesterolemia [1,5].

Data suggest that caloric intake is increasing among all ethnicities, age and socioeconomic groups, and diets contain more energy-dense, nutrient-poor foods [6]. Evidence also suggests that consumption of foods from fast-food restaurants, which typically serve foods high in total and saturated fat, has doubled during the past 30 years [7]. Consumption of unhealthy foods may be replacing that of fruits and vegetables (FV), which are known to provide essential vitamins, minerals and other nutrients [6]. Even though high levels of whole grain consumption have been linked to reduced rates of CVD and colon cancer, most adults do not consume enough of these foods [8]. Alcohol represents another dietary component that is associated with CVD risk factors. While moderate consumption of alcohol is protective against CVD, high levels of consumption are correlated with obesity, CVD and all-cause mortality [9]. Also, evidence regarding the readiness to change dietary habits among adults also indicates that most adults are either not aware or not prepared to change their current dietary habits thus adding a layer of difficulty in trying to promote healthful eating habits [10]. Given the strong link between diet and disease [11], it is important to evaluate and address dietary behaviors and readiness for change at a young age for effective prevention of CVD and related co-morbidities.

University marching band, dance team and cheer squad members perform physical activity for prolonged periods of time. It has been estimated that these athletes exercise at an intensity of 4.5–6 metabolic equivalents (METs) [12]. This MET category also includes activities such as tennis, recreational swimming, golfing, bicycling and lawn mowing. Even though these band members perform intense physical activity, they represent a largely unstudied population in terms of physical characteristics, fitness levels, dietary habits, prevalence of overweight and CVD risk factors. Their dietary habits and physical fitness could be of vital importance to their well-being and performance, especially in an environment of increased heat and humidity.

The primary purpose of this study was to assess prevalence of CVD risk factors, measure dietary intake, readiness to change dietary intake and finally, examine the relationship between dietary intake and risk factors for CVD among members of an urban university marching band, dance team and cheer squad. We hypothesized that dietary factors such as low intake of grains, FV, dairy and high intake of alcohol are significantly associated with the CVD risk factors such as body mass index (BMI), waist to hip ratio (WHR), blood pressure, blood glucose and blood cholesterol.

## Methods

This study examined baseline pre-season data collected in fall of 2004. This baseline analysis was part of the main study, the primary aim of which was to promote healthy eating habits and physical fitness for the prevention of obesity-related conditions and CVD among marching band, dance team and cheer squad members in an urban university (Bush et al., in preparation).

### Participants

Members of a mid-sized university marching band located in the southwest part of the United States (N = 275) participated in the study. The study was approved for the use of human subjects by the university Committee for the Protection of Human Subjects. The study investigators attended practice sessions during pre-season to recruit volunteers for the study. They informed the band members of the purpose of the study and the study procedures. During this time the investigators asked all interested band members to provide their contact information and review the informed consent document. Incentives were offered to the participants in the form of free fitness testing and nutrition counseling as a part of the study. The response rate was 93%. Informed consent was obtained from all participants prior to start of the study. Band members were also informed that they could refuse to partake in any portion of the testing procedures without negative consequences. During the pre-season, which is a 2-week band camp, participants attended a testing session in the investigator's laboratory. Participants refrained from any regular exercise programs two days prior to the testing by not participating in band activities during that period. They completed a self-administered demographic, nutrition and medical history questionnaire. Since 232 participants completed the nutrition survey, the final sample for the present study utilized for the analysis was N = 232. Please note that detailed description of the study protocol is presented elsewhere [13]. All data collection discussed below was performed on campus by the investigators and trained personnel.

### Anthropometrics

Body height (cm) to the nearest 0.5 cm was obtained using a standard physician's scale height stadiometer (Detecto, Creative Health Products, Inc., Plymouth, MI). Body weight (kg) was measured to the nearest 0.1 kg using a digital scale (Seca Alpha 770 digital scale). Body height and weight were used to calculate BMI. BMI was classified using the CDC classification as normal (18.5–24.9), overweight (25.0–29.9), and obese (≥ 30.0) [14]. Waist and hip circumference were obtained to the nearest centimeter to calculate WHR. BMI and WHR were used as markers for weight status. For WHR, we utilized the cutoff of 0.8 for females and 0.95 for males that has been recommended for identifying persons at high risk for CVD [15]. Physical characteristics of the participants are presented in Table 1.

**Table 1:** Characteristics of the study participants (N = 232).

**Variable**	**Total N = 232**	**Male N = 140**	**Female N = 92**	**p-value**
	**Mean **± **SD**	**Mean **± **SD**	**Mean **± **SD**	

Age (yr)	19.3 ± 1.5	19.4 ± 1.5	19.1 ± 1.6	0.217
Body weight (lbs)	168.1 ± 46.8	179.9 ± 43.5	150.2 ± 46.2	0.000*
Body height (cm)	170.9 ± 8.3	175.6 ± 6.1	163.9 ± 6.0	0.000*
Body Mass Index	26.1 ± 6.6	26.6 ± 6.3	25.3 ± 6.9	0.1604
Waist to Hip ratio	0.81 ± 0.07	0.83 ± 0.06	0.77 ± 0.07	0.0000*

Data are presented as mean ± SD and Frequency (N), percent (%)

* p < 0.05 significant difference between genders.

### Blood pressure, fasting blood glucose and total cholesterol

After resting for 10 minutes quietly, systolic and diastolic blood pressure (BP; mmHg) was obtained using a digital automatic blood pressure monitor (IntelliSense™, Omron, Healthcare Inc., Vernon Hills, IL). Fasting blood glucose (mg/dL) and total cholesterol (mg/dL) were obtained from each participant through a fingerstick puncture. The whole blood was analyzed for total blood cholesterol and blood glucose. Blood glucose values are presented as normal (≤ 110 mg/dL) or high (>110 mg/dL) [16]. Blood total cholesterol values are presented as normal (≤ 200 mg/dL) or high (>200 mg/dL) [17]. Systolic BP is presented as normal (≤ 140 mmHg) or high (>140 mmHg); and diastolic BP is presented as normal (≤ 90 mmHg) or high (>90 mmHg) [18]. Blood pressure, blood glucose and total cholesterol values are presented in Table 2.

**Table 2:** Prevalence of cardiovascular disease risk factors of study participants (N = 232).

**Variable**	**Total N (%)**	**Male (N = 140) N (%)**	**Female (N = 92) N (%)**	**p-value**
^§^Body Mass Index				
- Normal (18.5 – 24.9 kg/m2)	128 (55.0)	66 (47.5)	62 (67.4)	0.004*
- Overweight (25.0 – 29.9)	57 (24.7)	44 (31.7)	13 (14.1)	
- Obese (>30.0)	46 (19.9)	29 (20.9)	17 (18.5)	
Waist-to-Hip Ratio				
- <0.80	115 (49.6)	50 (35.7)	65 (70.0)	0.000*
- 0.80 – 0.94	108 (46.6)	84 (60.0)	24 (26.1)	
-≥ 0.95	9 (3.9)	6(4.3)	3 (3.9)	
Fasted Blood Glucose				
- Normal (≤ 110 mg/dl)	214 (92.2)	130 (92.9)	84 (91.3)	0.665
- High (>110 mg/dl)	18 (7.8)	10 (7.1)	8 (8.7)	
Fasted Blood Cholesterol				
- Normal (≤ 200 mg/dl)	208 (89.7)	128 (91.4)	80 (87.0)	0.274
- High (>200 mg/dl)	24 (10.3)	12(8.6)	12 (13.0)	
Systolic Blood Pressure				
- Normal (≤ 140 mmHg)	198 (85.3)	115 (82.1)	83 (90.2)	0.089
- High (>140 mmHg)	34 (14.7)	25 (17.9)	9 (9.8)	
Diastolic Blood Pressure				
- Normal (≤ 90 mmHg)	212 (91.4)	125 (89.3)	87 (94.6)	0.161
- High (>90 mmHg)	20 (8.6)	15 (10.7)	5 (5.4)	

Data are presented as total number (N) and percent (%) of total number in each category.

* Chi-square p < 0.05 significant difference between genders.

Abbreviations: BMI, body mass index; WHR, waist-to-hip ratio

^§^The sample size for BMI does not add to N = 232 secondary to some of the participants not having completed the measurements.

### Dietary habits

Participants (N = 232) completed a self-administered cross-sectional survey evaluating the number of servings of various foods eaten per day based on the recommendations of the 2000 USDA Food Guide Pyramid [19] as well as dietary stages of change [20]. The survey consisted of questions regarding the participants' eating behavior and dietary Stages of Change (pre-contemplation, contemplation, preparation, action and maintenance) using the Transtheoretical Model (TTM) [20] for all the food groups mentioned above. This allowed for assessment of the participants' readiness to make dietary changes. The TTM has been validated in previous studies as an effective method of evaluating readiness to change dietary behaviors in adults [21-23]. Our survey instrument was developed from these studies which indicate that self-assessment of diet using standard staging questions can be accurate reflections of intake. Staging questions were designed to categorize participants regarding their readiness to change according to the TTM. Participants were staged according to responses to the following questions: 1) How many servings of __________ (e.g., food group variable) do you usually eat in a day? 2) Have you been eating this amount for more than 6 months? 3) In the future do you intend to increase this amount, decrease this amount, or stay the same? And 4) do you intent to make this change in the next 6 months?

The survey instrument also contained one question regarding their alcohol consumption: Do you drink alcohol? Subjects who answered yes on alcohol intake were asked about the number of servings of alcohol consumed per week.

Pilot testing and focus groups affirmed understanding of question terminology in the survey and did not report any problems with the determination of what their perception of a serving was for the various food groups. Data from surveys were double entered and cross checked with error rates of <1%.

### Statistical analysis

Unpaired t-test and Pearson's chi square test were used to determine baseline differences in participant characteristics by gender and ethnicity. Data were also analyzed to determine if the participants who did not complete the nutrition survey were different in their CVD risk factor characteristics than those who did. Multiple linear regression analysis was used to determine the association between dietary intake of various food groups such as grains, meats, fruits & vegetables, dairy, water and alcohol (independent variables) and risk factors for CVD namely BMI, blood glucose, blood total cholesterol, and blood pressure (dependent variables). All CVD risk factors were used as continuous variables in the analysis. Bivariate and multivariate analysis was performed with each CVD risk factor as the outcome variable and each food group as the predictor variable. Covariates adjusted for in the multivariate analysis included age, gender, BMI and ethnicity. Results presented are those from the regression models that were statistically significant.

Staging for grains, FV, meats, dairy and water was determined using a staging algorithm. While the nutrition survey provided information on all five Dietary Stages of Change categories, due to small cell sizes it was necessary to collapse the stages of change from five stages to two stages. These were named the 'cognitive stage' (pre-contemplation, contemplation and preparation) since these participants have not made any overt behavior changes and; the 'behavior stage' (action and maintenance) since these participants have either made overt behavior changes for less than or more than 6 months and the behavior change is observable. Significance level for all analysis was set at p < 0.05.

## Results

### Physical characteristics and indicators of cardiovascular risk

Table 1 shows the baseline physical characteristics of the study participants. The participants (mean ± SD) were 19.3 ± 1.5 yrs and had a BMI of 26.1 ± 6.6. Sixty-five percent and 35% of the study population were White and non-White, respectively. Table 1 shows significant gender differences for body weight, body height and WHR. Overall, males had a higher WHR compared to females.

Table 2 shows the indicators of CVD risk for the study participants, subdivided into the categories of risk threshold for BMI, WHR, blood glucose, blood total cholesterol, and systolic and diastolic BP. The values are presented as the number of participants expressing the risk factor value. Overall, 45.0% of the participants were overweight or obese, 7.8% of had high blood glucose, 10.3% had high blood total cholesterol, 14.7% had high systolic blood pressure and 8.6% had high diastolic blood pressure. Results showed that 30.0% of the females had a WHR ≥ 0.80 and 4.3% of the males had WHR ≥ 0.95. It is important to note here that when data using the larger sample (N = 275 participants) was examined, results showed a significantly higher prevalence of elevated blood glucose (13.6%) and elevated blood cholesterol (22.6%) as compared to the 232 participants used in the present study. The prevalence of overweight and obesity, WHR and elevated blood pressure were not significantly different between the two samples.

### Dietary habits

Table 3 presents the dietary habits of the participants. The food categories analyzed for number of servings per day are grains, meats, FV, and dairy, water and alcohol intake. Greater than 70% of the participants consumed less than 6 servings of grains and drank less than 8 glasses of water per day. Eighty-three percent of the participants consumed less than 5 servings of FV per day. Although 99 participants responded positively to consuming alcohol; 157 participants (58%) reported the number of servings of alcohol consumed. Of the 157 participants, 73.2% reported consuming 1–2 servings of alcohol per week, 16.6% reported between 3 to 4 servings per week and 10.2% of participants reported 5 or more servings of alcohol per week. Overall, a higher percentage of females ate the recommended servings of grains, FV, dairy and water as compared to males; however, these differences were not statistically significant.

**Table 3:** Dietary habits of study participants: Number of servings of foods consumed per day (N = 232).

**Food Groups**	**Frequency N (%)**	**Males (N = 140) N (%)**	**Females (N = 92) N (%)**	p-value*
^§^Grains				
0 to 5 servings/day	156 (70.9)	100 (75.8)	56 (63.6)	0.088
6 to 11 servings/day	60 (27.3)	29 (22.0)	31 (35.2)	
>11 servings/day	4 (1.8)	3 (2.8)	1 (1.1)	
^§^Meats				
0 to 2 servings/day	57 (25.2)	34 (25.0)	23 (25.6)	0.993
3 to 4 servings/day	114 (50.4)	69 (50.7)	45 (50.0)	
>4 servings/day	55 (24.3)	33 (24.3)	22 (24.4)	
^§^Fruits and Vegetables				
0 servings/day	13 (5.6)	11 (7.9)	2 (2.2)	0.062
1 to 4 servings/day	178 (77.4)	109 (77.9)	69 (75.0)	
5 to 9 servings/day	38 (16.5)	19 (14.3)	20 (22.8)	
> 9 servings/day	1 (0.4)	N/A	N/A	
^§^Dairy				
0 servings/day	17 (7.4)	10 (7.3)	7 (7.6)	0.382
1 to 2 servings/day	101 (43.9)	64 (46.4)	37 (40.2)	
3 to 4 servings/day	91 (39.6)	49 (35.5)	42 (45.7)	
>4 servings/day	21 (9.1)	15 (10.9)	6 (6.5)	
^§^Water				
0 to 7 glasses/day	168 (72.7)	103 (74.1)	65 (70.7)	0.565
>7 glasses/day	63 (27.3)	36 (25.9)	27 (29.4)	
^≠^Alcohol (N = 157)				
1–2 servings per week	115 (73.3)	64 (69.6)	51 (78.5)	0.453
3–4 servings per week	26 (16.6)	17 (18.5)	9 (13.9)	
>= 5 servings per week	16 (10.2)	11 (11.9)	5 (7.7)	

Data are presented as frequency (N) and percent (%) of total number in each category.

* Chi-square p < 0.05 significant difference between genders.

N/A – not applicable. For fruits and vegetables, the >9 servings/day group was collapsed with 5 to 9 servings/day group due to small cell size.

^§ ^The sample size for these variables does not add to N = 232 secondary to some of the participants not answering all the questions on the nutrition survey.

^≠^157 (92 Males, 65 females) participants reported the number of servings of alcohol consumed per week.

### Dietary stages of change

Table 4 presents the dietary stages of change of the participants for various food groups. Results indicated that for grains, FV, and water, greater than 70% of the participants were in the cognitive stage, while for meats and dairy, 49.8% and 58.7%, respectively, were in the cognitive stage. Thus, the majority of the participants were *not *in an action or maintenance stage for fruits, vegetables, dairy and water consumption.

**Table 4:** Dietary Stages of Change of various food groups for study participants.

Food Groups*	**Frequency (N)**	**Percent (%)**
Grains (N = 191)		
Cognitive stage?	134	70.1
Behavior stage§	57	29.9
Meats (N = 219)		
Cognitive stage	109	49.8
Behavior stage	110	50.2
Fruits and Vegetables (N = 217)		
Cognitive stage	170	82.1
Behavior stage	37	17.9
Dairy (N = 218)		
Cognitive stage	128	58.7
Behavior stage	90	41.3
Water (N = 225)		
Cognitive stage	163	72.4
Behavior stage	62	27.6

Data are presented as frequency (N) and percent (%) of total number in each category.

≠Cognitive stage: pre-contemplation, contemplation, and preparation

§Behavior stage: action and maintenance

* The sample size for these variables does not add to N = 232 secondary to some of the participants not answering all the questions on the nutrition survey.

### Association of diet and indicators of CVD risk

Multiple linear regression analysis was performed to determine if the dietary intake of the various food groups such as grains, FV, meats, dairy as well as alcohol and water intake were significant predictors of the CVD risk factors namely BMI, systolic blood pressure, diastolic blood pressure, blood cholesterol and blood glucose. Multivariate analysis after adjusting for confounders such as age, gender and ethnicity showed that higher grains intake was positively associated with higher BMI (Adjusted β = 1.97, p = 0.030, 95% CI: 0.19, 3.74) and higher alcohol intake was also positively associated with higher BMI (Adjusted β = 0.15, p = 0.002, 95% CI: 0.06, 0.24). Other associations of interest between the food groups and CVD risk factors were not statistically significant.

## Discussion

Overall a high percentage of participants reported eating fewer grains, FV and drinking fewer glasses of water than recommended. Additionally, a majority of the participants were not ready to produce a change in their eating behaviors. Our results also identified a positive association between grain consumption, alcohol intake and BMI.

Forty-five percent of the participants were overweight or obese. The high prevalence of overweight/obesity that was observed in our study concurs with other studies on college-age students which report that 20% to 40% of college students are overweight or obese [24,25]. The 2006 National College Health Assessment (NCHA) survey on 23,863 students reported that 31% of the U.S college students were overweight or obese based on self-reported height and weight [25]. This lower prevalence of overweight in the NCHA statistics as compared to our study could be related to the ethnic distribution since 77% of the NCHA population was Caucasian as compared to 65% in our study; and Caucasians have a lower prevalence of obesity as compared to Hispanics of African Americans [26]. Secondly, our study measured height and weight on all the participants. Usually in self-report, as done in NCHA, height is over-reported and weight is under-reported resulting in a lower estimate of BMI [27]. The high percentage of females with a WHR ≥ 0.80 indicates an increased risk for development of atherosclerotic plaque and CVD in these participants [28]. This high prevalence of overweight in these athletes, especially the females is quite concerning since it puts this population of athletes at increased risk for CVD. Also, given the rigorous training that these athletes often have to do under extreme weather conditions, it is important that they are in peak physical shape for optimal performance. These results warrant the need for design and implementation of targeted obesity-prevention programs for females in this population.

When compared to the 2000 Dietary Guidelines for Americans, our data indicate that less than 20% of participants in this urban marching band university were consuming 5 or more servings of FV per day. Similar results were observed in the 2006 NCHA survey, which reported that only 6.2% of the students were usually consuming 5 or more servings of FV a day [29,30]. Also, more than 70% of the participants were not consuming the recommended servings for grains. The newer 2005 Dietary Guidelines for Americans [19] recommend 2 cups (4 servings) of fruits and 2.5 cups (5 servings) of vegetables, 5.5 ounces (2 servings) for meats and, 6-ounce equivalents (6 servings) of grains (of which 3 ounces are whole grains) per day at the 2000 calorie level. When compared to these newer guidelines, majority of our study participants are not meeting the recommendations for FV and grains and, are consuming more than the recommended amount for meats. Unfortunately, the current survey did not capture the total caloric intake to determine the participants' intake of various foods relative to the total calories. Also no data was collected on whole grain intake which would have clinical implications since a diet high in low fat dairy, whole grains, and FV along with exercise is considered to be protective against several chronic diseases including obesity, CVD, and cancer [29,30]. The positive association between grains intake and BMI seen in our study could be suggestive of a high intake of simple carbohydrates which have been associated with overweight and obesity [31]. It would be worthy for future studies to collect data on whole grain consumption and examine their relationship with CVD risk factors in this population of student athletes.

The results from the Dietary Stages of Change assessment suggest that the majority of participants are in the cognitive stages for the dietary intake of grains and FV indicating that they are not making any overt changes towards changing their eating behavior for grains and FV. These data imply that health promotion programs aimed at moving these athletes from the cognitive stages to the behavior stages by increasing knowledge and providing necessary skills to improve dietary habits are critical in this population. These data provide critical information regarding the study population's readiness to change their dietary habits which clearly indicates that, at this time, it would be counterproductive to provide action-oriented material about healthy dietary change behaviors. In fact, the focus needs to be more on raising awareness of poor dietary habits, pros of eating more whole grains, FV to move the participants towards action. It is also important to note that the Stages of Change model has potential for public health interventions as well as individual nutrition interventions by health care practitioners towards improving the dietary habits of this population. Additionally, environmental factors, such as availability of FV and whole grain foods on-campus, need to be evaluated. Psychosocial factors such as knowledge, attitudes, beliefs, self-efficacy, outcome expectations and social support influencing diet and weight status in these athletes also need to be assessed.

Greater than fifty-seven percent of the participants indicated consuming 1 or more servings of alcohol per week with more than 10% of them drinking 5 or more servings of alcohol per week. These results concur with the NCHA statistics which reported that 70% of college students used alcohol in the last 30 days [25]. While moderate consumption of alcohol is protective against CVD [32], high consumption has adverse effects on health [33]. Alcohol is detrimental to athletic performance since it is a diuretic, gives problems with heat regulation, causes hypoglycemia, reduces reaction-times, endurance and increases risk for injury [34]. Our study also found a positive association of alcohol intake and BMI. This concurs with the literature that shows frequent consumption of alcohol is associated with weight gain given its high caloric content [35]. Also, given that the average age of the study participants was 19 years; our results suggest a high prevalence of underage drinking in this population. Unfortunately, midcourse reviews of the Healthy People 2010 and Healthy Campus 2010 have shown no change nationally in alcohol use and abuse [36,37]. We strongly recommend using evidence-based programs that are intentionally and cautiously researched, planned, implemented and evaluated in college campuses, especially among athletes. Using health marketing, ecological/environmental, policy and social marketing approaches could potentially work to influence positive health behaviors of students. Additionally, existing alcohol and substance abuse awareness programs need to be evaluated.

Perhaps the most alarming finding of our study was the high percentage of participants with high WHR, elevated blood glucose, cholesterol and systolic and diastolic blood pressure. The major risk factors for CVD include levels of cholesterol (total cholesterol, low-density lipoprotein, and high density lipoprotein), BMI, blood pressure, cigarette smoking and diabetes [17,38]. Studies have shown that total cholesterol have similar predictive values as LDL for CVD secondary to the atherogenic properties of cholesterol [39]. The European SCORE project advocates the use of total cholesterol in isolation [40]. However, recent literature has identified TC/HDL or LDL/HDL ratios having superior predictive values with respect to CVD [38]. Thus, it is a limitation of the current study that LDL, HDL values were not determined and it would be worthy for future studies to examine these measures.

These results could indicate the presence of components of metabolic syndrome among participants which is considered to be a risk factor for CVD [41]. Huang et al. [41] examined the prevalence of metabolic syndrome among 163 college students. They concluded that 27% of the population was overweight and 11.7% had high blood cholesterol [41]. Our study did not assess the prevalence of metabolic syndrome in this population which would have provided a greater insight into the health status and CVD risk among this population of collegiate athletes.

### Strengths and limitations

This study is among the first to evaluate the dietary habits and examine their relationship to CVD risk factors in marching band, dance team and cheer squad members, dance team and cheer squad. The large sample size lends sufficient power to the study. Currently there is little data available on the dietary habits as well as CVD risk factors in this population. While not confirmatory, these results are an appropriate starting point for further in-depth exploration into these relationships between diet and CVD risk factors in student athletes and also pave the way for developing future research and health promotion/health education programs targeting this population.

These strengths notwithstanding, our study has its limitations. We had a high response rate since the study was conducted on campus and the participants had incentives in the form of free nutrition and fitness counseling sessions conducted by exercise physiologists and licensed dietitians which can be very expensive if the participants were to obtain these on their own. This high response rate could have biased our results away from the null. Secondly, only about 84% (N = 232) of the total respondents completed the nutrition survey. Also, it appears that the prevalence of the CVD risk factors was higher in the larger sample (N = 275) as compared to those who did not complete the survey which could have biased our results towards the null. However, all attempts were made by study investigators to contact the participants who did not complete the nutrition survey.

Another limitation is that this study relied on self-reported measures of dietary intake which could bias the validity [42]. However, these measures are commonly used in epidemiologic studies with reasonable accuracy [43]. Also, no data was collected on intake of fast foods or fat intake, both of which have been shown in studies to be consumed at high levels among children, adolescents and college students [44]. While the analysis was stratified on gender, it was not stratified by type of sport. This was primarily because majority of the sample were band members with not sufficient number of participants in the other sports to detect significant differences by sport. Of the 31 total cheerleaders and dance members (N = 14 cheerleaders and N = 17 dance members) who consented to participate in the study, only seven responded to the nutrition survey. Since cheerleading and dance are considered a 'weight-conscious' sport as compared to participating in the band, collapsing the three together could probably have biased the results towards the null. However, given the small sample of cheerleaders and dance members (N = 7) in the present study, this is highly unlikely. Finally, given the cross-sectional nature of our study, causality cannot be established and since this was secondary data analysis, the analysis is exploratory.

## Conclusion

In sum, our results suggest a trend toward significant health risks in this elite members of a marching band unless an intervention of diet and exercise are performed. A high percentage of members of this urban marching band have a poor dietary intake and demonstrate a relatively high prevalence of overweight, hyperglycemia, hypercholesterolemia, and hypertension that increase their risk for CVD as well as other lifestyle-related illnesses. These results are especially important since these athletes participate in rigorous activities often under extreme weather conditions throughout the season and need to be in sound health for optimal performance. The unhealthy eating behaviors and a relatively high prevalence of alcohol consumption identified in the current study imply the need to evaluate existing health promotion programs aimed at improving the lifestyle of college students on campus and for evidence-based programs to be implemented. Finally, ACHA tools such as Standards of Practice for Health Promotion in Higher Education [45] and Healthy Campus 2010 [37] should be used to develop, test and implement on-campus health promotion programs geared towards improving the dietary habits and reducing the prevalence of CVD risk factors in this population.

## Competing interests

The authors declare that they have no competing interests.

## Authors' contributions

SVS conceptualized the study question and design, conducted statistical analysis, interpretation of results, drafting of manuscript and reviewing of the manuscript. JAB is the Principal investigator of the study, conducted data collection, drafting and critically evaluating the manuscript. AJL did data collection, data entry, drafting of the manuscript and reviewing the manuscript. MK did data collection and critically reviewed the manuscript. DA did data collection and critically reviewed the manuscript. GB did data collection and critically reviewed the manuscript. DB participated in study design and data collection.

## Consent

Informed consent was obtained from all participants prior to the study and the study protocol was approved by the University of Houston, Committee for Protection of Human Subjects.
